# A Quantum Computing Method for AC Power Flow with Residual-Controlled Dynamic Precision

**DOI:** 10.3390/e28090988

**Published:** 2026-09-03

**Authors:** Mengbo Yan, Dabo Zhang, Kanghai Yang, Yuan Cao

**Affiliations:** Anhui Provincial Laboratory of Renewable Energy Utilization and Energy Saving, Hefei University of Technology, Hefei 230009, China; zhangdb2004@163.com (D.Z.); 2025170562@mail.hfut.edu.cn (K.Y.); 2025110321@mail.hfut.edu.cn (Y.C.)

**Keywords:** quantum singular-value transformation, ill-conditioned Jacobian matrix, Tikhonov regularization, dynamic quantum precision

## Abstract

To address the amplification of correction errors caused by an ill-conditioned AC power-flow Jacobian under heavily loaded operating conditions, as well as the excessive circuit depth associated with a fixed quantum-solution accuracy, this paper proposes a residual-controlled, regularized quantum singular-value transformation (QSVT) method within an inexact Newton power-flow framework. First, the power-mismatch vector and state variables are scaled, and the Newton correction is reformulated as a Tikhonov-regularized least-squares subproblem. A bounded regularization filter is then approximated using Chebyshev polynomials, allowing QSVT to directly transform the singular values of the Jacobian matrix. On this basis, the residual of the linear subproblem associated with the quantum-approximate correction is defined, and constraints are established to relate the polynomial-approximation error, block-encoding error, and quantum measurement error to the inexact Newton forcing term. The QSVT polynomial degree and quantum-solution accuracy are subsequently adjusted dynamically according to the outer power-flow residual. Theoretical analysis establishes the boundedness of the regularized correction, a sufficient descent condition for the quantum-approximate correction, and the relationship between the solution error and cumulative query complexity under dynamically controlled quantum accuracy.

## 1. Introduction

Power-flow analysis characterizes the nonlinear relationship between specified network topology, component parameters, and nodal power injections and the resulting steady-state bus voltages and branch power flows. It therefore constitutes a fundamental computational tool for power-system planning studies, operational analysis, and security assessment. The Newton–Raphson and fast-decoupled methods have established the principal numerical frameworks for deterministic AC power-flow analysis [1,2]. However, the increasing penetration of renewable energy resources, energy storage systems, and controllable loads has introduced pronounced temporal variability and stochasticity into power-system operation. Consequently, power-flow analysis is evolving from the solution of a single deterministic operating condition toward repeated computations over time-series data, probabilistic distributions, and large-scale scenario sets [3,4,5]. Under this paradigm, the overall computational burden is determined not only by the cost of an individual power-flow solution, but jointly by the network size, the number of scenarios, and the numerical convergence difficulty associated with each scenario.

The numerical robustness of AC power-flow analysis is governed primarily by the spectral properties of the Jacobian matrix. As the system approaches a heavily loaded operating point, a saddle-node bifurcation, or the voltage-stability boundary, the smallest singular value of the Jacobian matrix may decrease substantially. Consequently, the Newton correction becomes increasingly sensitive to power mismatches, model perturbations, and round-off errors, potentially resulting in excessively large steps, iterative oscillations, or even divergence [6,7,8,9]. This issue is particularly pronounced in distribution networks, where high resistance-to-reactance ratios, radial or weakly meshed topologies, and phase imbalance weaken the conventional active-power–phase-angle and reactive-power–voltage-magnitude decoupling relationships while exacerbating scaling disparities among state variables [10,11]. Therefore, multi-scenario analysis and ill-conditioned linearization are not independent computational challenges: the former increases the cumulative cost of solving the linear subproblems, whereas the latter raises the stabilization and accuracy requirements of each individual solve.

Classical approaches for enhancing the robustness of ill-conditioned power-flow calculations can be broadly categorized into globalization, regularization, and inexact-solution strategies. The optimal multiplier method enlarges the convergence region by adaptively adjusting the Newton step length, whereas continuation power flow introduces a continuation parameter and employs a predictor–corrector procedure to trace the steady-state solution branch near critical operating points. The Levenberg–Marquardt method incorporates a damping term into the least-squares model to interpolate between the Gauss–Newton and gradient directions [12,13], while Tikhonov regularization suppresses error amplification in ill-conditioned components by limiting the gain associated with small-singular-value directions [14,15]. Inexact Newton methods further use a forcing term to control the relative residual of the linear subproblem, thereby matching the inner-solver accuracy to the progress of the outer nonlinear iteration and avoiding oversolving during the early stages [16,17,18]. Although these methods can enlarge the convergence region and improve numerical stability, their computational kernels still rely on repeated classical sparse linear-algebra operations. Moreover, when a regularized least-squares problem is solved through the normal equations, the two-norm condition number of the Jacobian is effectively squared, thereby further aggravating the underlying ill-conditioning.

Quantum linear algebra provides an implementation paradigm for the aforementioned computational kernel that differs fundamentally from classical matrix factorization methods. The HHL algorithm and its subsequent extensions have shown that, under assumptions concerning matrix sparsity, bounded condition numbers, quantum-state preparation, and solution readout, a quantum state proportional to the solution of a linear system can be prepared, with improved dependence on solution precision or the matrix-access model [19,20,21,22]. Block encoding, qubitization, and quantum singular-value transformation further unify the implementation of matrix functions within a framework of polynomial singular-value transformations applied to embedded unitary matrices, enabling matrix inversion, pseudoinversion, and regularized spectral filtering [23,24,25,26,27]. Unlike approaches based on eigendecomposition, QSVT acts directly on singular values. It can therefore accommodate nonsymmetric Jacobian matrices and implement Tikhonov-type filtering without explicitly forming the product of the Jacobian transpose and the Jacobian itself, thereby providing a theoretical basis for avoiding the squared growth in the condition number induced by the normal equations.

Existing studies on quantum power flow have primarily focused on quantum linear-system formulations of fast-decoupled power flow, validation on real quantum hardware, hybrid quantum–classical implementations of DC power flow, and quantum-neural-network surrogate models [28,29,30,31], with recent extensions to stochastic power flow and operational risk assessment [32]. In practical AC power-flow analysis, however, the Jacobian matrix is updated continuously with the iterative state, is generally nonsymmetric, and may become severely ill-conditioned near critical operating regions. The corresponding quantum-resource requirements are therefore determined not only by the system dimension, but also by the condition number, block-encoding cost, target accuracy, postselection success probability, and classical readout requirements. Recent end-to-end complexity analyses have further demonstrated that the potential advantage of quantum power-flow algorithms cannot be assessed independently of the matrix condition number, input-access model, and output format [33]. The representative studies reviewed above have not yet established an accuracy allocation mechanism for successive nonlinear power-flow iterations. Applying a uniformly high-precision QSVT circuit at every iteration would incur unnecessary quantum-resource costs while the local linearization error remains dominant, whereas maintaining a fixed low precision throughout the solution process may cause quantum implementation errors to limit the local convergence rate and final solution accuracy near the power-flow solution.

To address these issues, this paper proposes a residual-controlled, regularized QSVT-based inexact Newton method for power-flow analysis. The main contributions are summarized as follows.

(1) Regularized quantum power-flow formulation. The state variables and power mismatches are nondimensionally scaled, and the Newton correction is formulated as a Tikhonov-regularized least-squares problem. QSVT is then applied to the scaled Jacobian matrix to implement a bounded regularized singular-value filter. By avoiding the explicit formation of the normal equations, the proposed formulation eliminates the associated condition-number squaring effect. It also suppresses error amplification in ill-conditioned components by limiting the filter gain along small-singular-value directions.
(2) Residual-driven dynamic quantum-precision configuration. The residual of the regularized linear subproblem is adopted as the error metric linking the outer power-flow iteration to the inner quantum solve. The QSVT polynomial-approximation error, block-encoding error, residual-state preparation error, measurement error, and norm-recovery error are incorporated into a unified error budget. Their admissible magnitudes are related to the outer power mismatch through an inexact Newton forcing term. Accordingly, the QSVT polynomial degree and measurement precision are dynamically adjusted according to the power-flow residual: relatively coarse precision is permitted during the early iterations, whereas the accuracy is progressively tightened as the solution is approached. This strategy reduces the cumulative quantum-resource cost while preventing quantum implementation errors from limiting the final convergence accuracy.

## 2. Regularized Inexact Newton Model for Ill-Conditioned Power Flow

This section begins with the AC power-flow equations and analyzes the error amplification of Newton corrections caused by an ill-conditioned Jacobian matrix. On this basis, a numerically stable power-flow correction model is constructed through variable scaling and Tikhonov regularization. An inexact Newton condition is then introduced to provide a unified error criterion for the subsequent QSVT-based approximate solution and dynamic-accuracy allocation.

### 2.1. AC Power-Flow Equations and Jacobian Ill-Conditioning

AC power-flow calculations can be formulated as a system of nonlinear equations. The state vector is typically composed of bus-voltage angles and voltage magnitudes, expressed as $x={\left[\theta^{T},V^{T}\right]}^{T}$, and the power-mismatch vector is defined as $F(x)=\left[\begin{array}{c} \Delta P(x)\\ \Delta Q(x) \end{array}\right]$, where ΔP and ΔQ denote the active- and reactive-power-mismatch vectors, respectively.

At the kth iteration, the Newton–Raphson method applies a first-order linearization to the power-flow equations, as given in (1):

(1) $$J_{k}\Delta x_{k}=-F_{k},$$

where

(2) $$F_{k}=F(x_{k}),J_{k}={\left.\frac{\partial F}{\partial x}\right|}_{x=x_{k}},$$

$J_{k}$ denotes the power-flow Jacobian matrix, and $\Delta x_{k}$ is the state correction vector. The state variables are updated according to (3):

(3) $$x_{k+1}=x_{k}+\Delta x_{k}$$

Let the singular-value decomposition of the Jacobian matrix be given by

(4) $$J_{k}=U_{k}\sum_{k}V_{k}^{T},$$

where

(5) $$\sum_{k}=diag(\sigma_{1,k},\sigma_{2,k},\ldots ,\sigma_{n,k}),$$

where $U_{k}$ is the orthogonal matrix whose columns are the left singular vectors of $J_{k}$, $\sum_{k}$ is the diagonal matrix containing the singular values, and $V_{k}$ is the orthogonal matrix whose columns are the right singular vectors of $J_{k}$.

When $J_{k}$ is nonsingular, the Newton correction can be expressed as

(6) $$\Delta x_{k}=-\sum_{i=1}^{n}\frac{u_{i,k}^{T}F_{k}}{\sigma_{i,k}}v_{i,k},$$

where $u_{i,k}$ denotes the component of the power mismatch along the corresponding left singular vector direction, $v_{i,k}$ represents the associated correction direction in the state-variable space, and $\sigma_{i,k}$ characterizes the amplification factor between these two directions. As indicated by the above expression, when the system approaches the voltage-stability boundary or operates under stressed conditions, some singular values of the Jacobian matrix may become close to zero, causing the corresponding components of the correction vector to be substantially amplified. Consequently, small perturbations in the power-mismatch vector, numerical errors in matrix computations, and quantum-approximation errors may all be converted into large state corrections, potentially leading to oscillatory iterations or convergence failure.

### 2.2. Dimensionless Scaling of State Variables and Power Mismatches

The voltage angles, voltage magnitudes, active powers, and reactive powers in the power-flow equations have different physical units and numerical ranges. If regularization is applied directly to the original Jacobian matrix, the effect of the regularization parameter may depend on the variable scales, and large numerical disparities among different columns of the matrix may further worsen its conditioning. To reduce these scale differences, a state-scaling matrix $S_{x}$ and a residual-scaling matrix $S_{f}$ are introduced. The scaled Jacobian matrix is defined as

(7) $$\tilde{J_{k}}=S_{f}J_{k}S_{x},$$

Accordingly, the original Newton correction equation is transformed into

(8) $$\tilde{J_{k}}s_{k}=-\tilde{F_{k}},$$

where $s_{k}$ denotes the dimensionless correction vector. The entries of matrix $S_{x}$ may be selected according to the characteristic variation ranges of the voltage magnitudes and phase angles, whereas those of matrix $S_{f}$ may be determined from the reference scales of the active- and reactive-power mismatches. The scaling operation does not alter the physical solution of the power-flow equations; it only provides a numerical reformulation of the linear correction equation.

### 2.3. Tikhonov-Regularized Power-Flow Correction Model

When the system is heavily loaded or operates near the voltage-stability boundary, $\tilde{J_{k}}$ may become severely ill-conditioned. Directly solving the corresponding linear system can therefore amplify residual and computational errors associated with small-singular-value directions. To suppress this amplification, the linear correction problem is reformulated as the following Tikhonov-regularized least-squares model:

(9) $$s_{k}^{*}=\underset{s\in {\mathbb{R}}^{n}}{\arg\min}\left[\frac{1}{2}{\left\|\tilde{J_{k}}s+\tilde{F_{k}}\right\|}_{2}^{2}+\frac{\mu }{2}{\left\|s\right\|}_{2}^{2}\right],$$

where s is the local optimization variable, $s_{k}^{*}$ denotes the corresponding minimizer at the kth iteration, ${\left\|\cdot \right\|}_{2}$ denotes the Euclidean norm of a vector, and μ is the Tikhonov regularization parameter.

The first term of the objective function measures the residual remaining after the correction vector is substituted into the linearized power-flow equations, whereas the second term penalizes the magnitude of the correction vector. A smaller value of μ produces a search direction closer to the standard Newton correction but provides weaker suppression of ill-conditioned components. A larger value of μ improves numerical stability at the expense of increased regularization bias. In this study, μ is not updated adaptively during the iterative process; instead, its value is selected through sensitivity analysis under different operating conditions.

It should be emphasized that a fixed positive regularization parameter does not change the exact power-flow solution itself. Since the regularized correction vanishes when F(x)=0, every nonsingular solution of the original power-flow equations remains a fixed point of the regularized iteration. The principal effect of μ is instead on the local iteration mapping and the correction direction. An excessively large value of μ suppresses the Newton correction more strongly and may therefore slow the asymptotic convergence, whereas an excessively small value provides weaker protection against ill-conditioned singular directions and quantum implementation errors. Under finite stopping tolerances and approximate quantum solves, an inappropriate value of μ may consequently increase the number of iterations or make the attainable residual more sensitive to implementation errors, even though it does not introduce a different exact power-flow root.

By imposing the first-order optimality condition on the objective function, the following regularized correction equation is obtained:

(10) $$\left({\tilde{J_{k}}}^{T}\tilde{J_{k}}+\mu I_{n}\right)s_{k}^{*}=-{\tilde{J_{k}}}^{T}\tilde{F_{k}},$$

where $I_{n}$ denotes the identity matrix of order n. To further illustrate the effect of regularization on the ill-conditioned directions, the scaled Jacobian matrix is decomposed using the singular-value decomposition given in (4). The regularized correction can then be expressed as

(11) $$s_{k}^{*}=-\sum_{i=1}^{n}\frac{{\tilde{\sigma }}_{i,k}}{{\tilde{\sigma }}_{i,k}^{2}+\mu }({\tilde{u}}_{i,k}^{T}{\tilde{F}}_{k}){\tilde{v}}_{i,k},$$

where the filter factor is defined as

(12) $$f_{\mu }(\sigma )=\frac{\sigma }{\sigma^{2}+\mu },$$

This function is referred to as the Tikhonov singular-value filter function. In the standard Newton correction, the corresponding singular-value mapping is 1/σ, which increases rapidly as σ approaches zero. By contrast, the regularized filter function tends to zero as the singular value approaches zero, thereby suppressing anomalously large corrections associated with small-singular-value directions. When the singular value σ is much larger than the characteristic regularization scale $\sqrt{\mu }$, the following approximation holds:

(13) $$f_{\mu }(\sigma )\approx \frac{1}{\sigma },$$

In this case, the regularized correction is close to the standard Newton correction. It follows that Tikhonov regularization primarily attenuates the correction components associated with ill-conditioned directions, while exerting only a limited influence on well-conditioned directions corresponding to larger singular values.

### 2.4. Inexact Newton Condition for Quantum-Approximate Corrections

In practical quantum computation, QSVT produces an approximate correction rather than the exact one, owing to errors arising from polynomial approximation, block encoding, quantum-state preparation, and measurement. Requiring the quantum computation to achieve high accuracy at every iteration would substantially increase the degree of the QSVT polynomial and the depth of the quantum circuit. Conversely, insufficient accuracy may impair the convergence of the outer power-flow iteration. Therefore, this study adopts an inexact Newton framework and uses the residual of the linear subproblem to determine whether the quantum-computed correction satisfies the accuracy requirement of the current iteration.

The residual of the quantum linear solve is defined as

(14) $$r_{k}^{q}=({\tilde{J_{k}}}^{T}\tilde{J_{k}}+\mu I_{n}){\hat{s}}_{k}+{\tilde{J_{k}}}^{T}\tilde{F_{k}},$$

where ${\hat{s}}_{k}$ denotes the approximate solution obtained using QSVT. When ${\hat{s}}_{k}=s_{k}^{*}$, $r_{k}^{q}=0$. The approximate quantum correction is required to satisfy ${\left\|r_{k}^{q}\right\|}_{2}\leq \eta_{k}{\left\|{\tilde{J_{k}}}^{T}\tilde{F_{k}}\right\|}_{2}$, where $\eta_{k}\in \left[0,1)\right.$ denotes the inexact Newton forcing term at the kth iteration. This condition does not require the quantum correction to coincide exactly with the exact solution; rather, it constrains the relative residual of the linear subproblem. During the early iterations, the power-flow state is relatively far from the exact solution, and the local linear model has limited accuracy. A larger value of $\eta_{k}$ may therefore be adopted to permit lower QSVT solution accuracy. As the power-flow residual decreases, $\eta_{k}$ should be reduced accordingly to improve the accuracy of the quantum correction and preserve convergence near the solution.

## 3. QSVT-Based Regularized Power-Flow Solution Method

This section further develops a quantum implementation of the regularized model. Rather than directly solving the normal equations, the proposed method does not explicitly construct ${\tilde{J_{k}}}^{T}\tilde{J_{k}}+\mu I_{n}$; instead, it applies the regularizing transformation directly to the singular values of the scaled Jacobian matrix $\tilde{J_{k}}$. This approach preserves the suppression of small-singular-value components provided by Tikhonov regularization while avoiding the squared condition number and increased matrix-operation cost associated with forming the normal equations.

### 3.1. Normalization and Block Encoding of the Scaled Jacobian Matrix

QSVT requires the singular values of the input matrix to lie within the interval $\left[0,1\right]$. Therefore, $\tilde{J_{k}}$ must first be spectrally normalized. Let $a_{k}\geq {\left\|{\tilde{J}}_{k}\right\|}_{2}$ denote the normalization factor, and define

(15) $$A_{k}=\frac{{\tilde{J}}_{k}}{a_{k}},$$

where ${\left\|\cdot \right\|}_{2}$ denotes the matrix spectral norm, i.e., the largest singular value; $a_{k}$ is the normalization factor at the kth iteration; and $A_{k}$ denotes the normalized Jacobian matrix.

In a quantum circuit, the matrix $A_{k}$ is not manipulated directly as a conventional numerical array; instead, it is embedded into a higher-dimensional unitary matrix. Let $U_{A_{k}}$ denote a block-encoding unitary that uses a ancillary qubits. If the following condition holds

(16) $${\left\|A_{k}-\left({\langle 0|}^{⊗a}⊗I_{n}\right)U_{A_{k}}\left({|0\rangle }^{⊗a}⊗I_{n}\right)\right\|}_{2}\leq \varepsilon_{BE,k}$$

where $U_{A_{k}}$ denotes an approximate block encoding of $A_{k}$; a is the number of ancillary qubits; $I_{n}$ is the identity matrix of order n; and $\varepsilon_{BE,k}$ denotes the block-encoding error at the kth iteration.

### 3.2. Regularized Singular-Value Filter Function

The function given in (12) directly characterizes how Tikhonov regularization treats different singular directions. Compared with the reciprocal mapping 1/σ used in the standard Newton method, the relative retention factor of the ith singular direction after regularization is given by

(17) $$q_{\mu }\left(\sigma \right)=\frac{f_{\mu }(\sigma )}{1/\sigma }=\frac{\sigma^{2}}{\sigma^{2}+\mu },$$

When the squared singular value $\sigma^{2}$ is much larger than the regularization parameter μ, $q_{\mu }\left(\sigma \right)$ approaches 1, indicating that regularization has little effect on the well-conditioned directions associated with larger singular values. When the squared singular value $\sigma^{2}$ is much smaller than the regularization parameter μ, $q_{\mu }\left(\sigma \right)$ approaches 0, indicating that corrections along small-singular-value directions are significantly attenuated. Therefore, $\sqrt{\mu }$ can be regarded as the characteristic scale of the regularization filter. For singular values above this scale, the correction remains close to the standard Newton direction; for singular values below this scale, the filtering effect progressively strengthens.

Regularization does not improve numerical stability without cost. Along the ith singular direction, suppressing the correction also leaves a certain portion of the linearized residual unresolved. Substituting the regularized correction into the linearized equation yields the residual-retention coefficient along each left singular direction as

(18) $$\gamma_{\mu }(\sigma )=\frac{\mu }{\sigma^{2}+\mu },$$

Moreover, this coefficient satisfies the following relationship with $q_{\mu }\left(\sigma \right)$:

(19) $$q_{\mu }(\sigma )+\gamma_{\mu }(\sigma )=1,$$

Therefore, regularization essentially represents a trade-off between reducing correction amplification and retaining part of the linearized residual. A larger μ improves stability under ill-conditioned operating conditions but leaves more residual to be resolved in the current iteration. A smaller μ yields behavior closer to that of the standard Newton method but provides weaker suppression of small-singular-value directions.

To transform $f_{\mu }(\sigma )$ into a bounded function that is implementable by QSVT, define the normalized regularization parameter as

(20) $$\gamma_{k}=\frac{\sqrt{\mu }}{a_{k}},$$

For the normalized singular value $z=\frac{\sigma }{a_{k}}\in \left[0,1\right]$, define the bounded regularization filter function as

(21) $$h(z)=\frac{2\gamma_{k}z}{z^{2}+\gamma_{k}^{2}},$$

This function is odd and satisfies $\left|h(z)\right|\leq 1,z\in \left[-1,1\right]$. It attains its maximum when $\left|z\right|=\gamma_{k}$. The original regularization filter function and the bounded function satisfy the following relationship:

(22) $$f_{\mu }(a_{k}z)=\frac{1}{2\sqrt{\mu }}h(z),$$

Therefore, the regularized correction can be equivalently expressed as

(23) $$s_{k}^{*}=-\frac{1}{2\sqrt{\mu }}h^{\left(\sigma \right)}(A_{k}){\tilde{F}}_{k},$$

Here, $h^{\left(\sigma \right)}(A_{k})$ denotes applying the function h to each singular value of $A_{k}$ while leaving the corresponding left and right singular vectors unchanged. The external coefficient $1/\left(2\sqrt{\mu }\right)$ restores the actual magnitude of the regularized correction, whereas the bounded function h performs the spectral filtering within the quantum circuit. Because h(0)=0, zero singular values do not cause the function to diverge, which is an important advantage of regularized filtering over directly approximating the reciprocal function.

**Figure 1:**
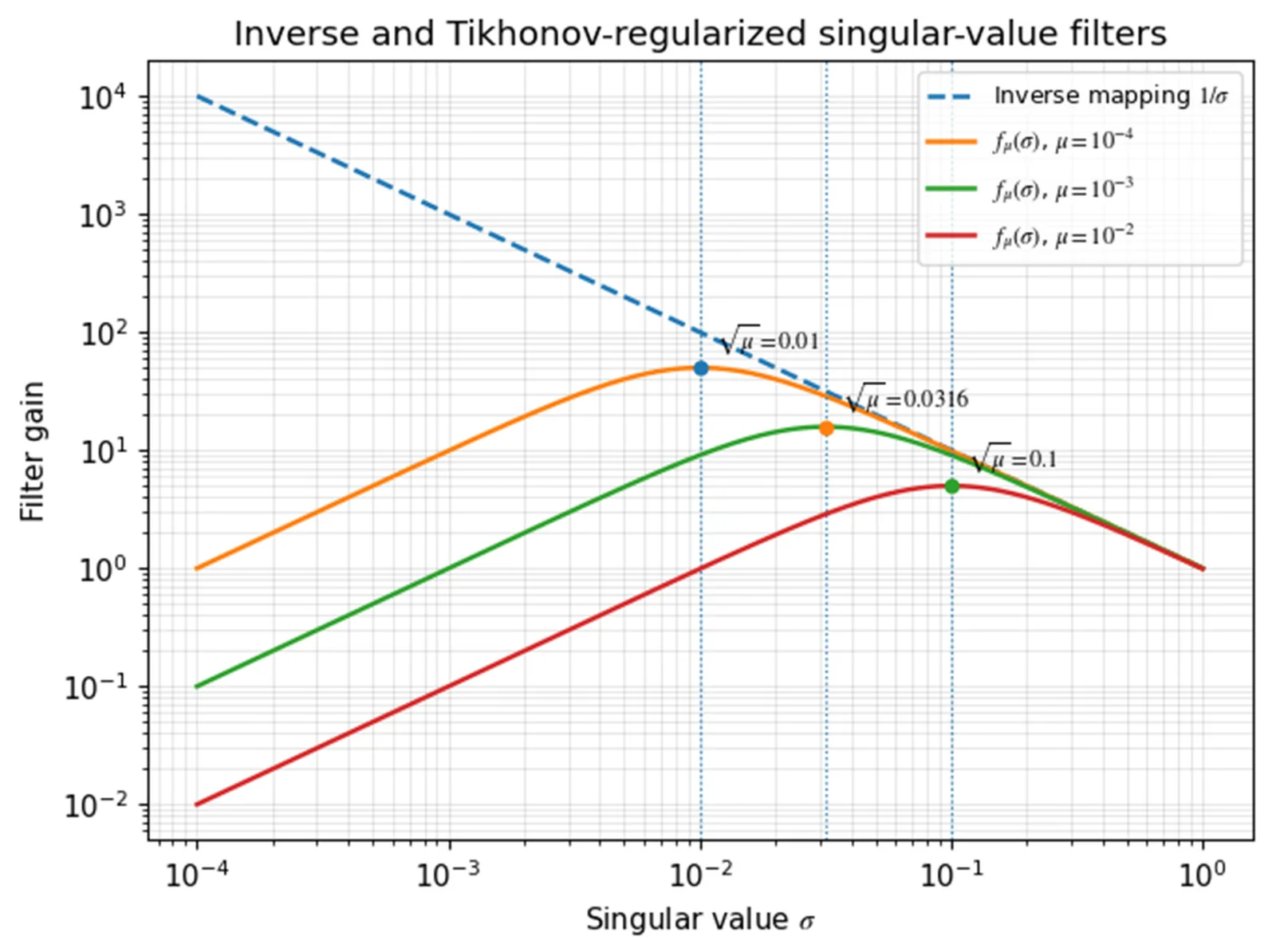
Gain curves of the inverse mapping and Tikhonov singular-value filters for different regularization parameters. Regularization bounds the gain associated with small singular values and suppresses error amplification in ill-conditioned directions.

**Figure 2:**
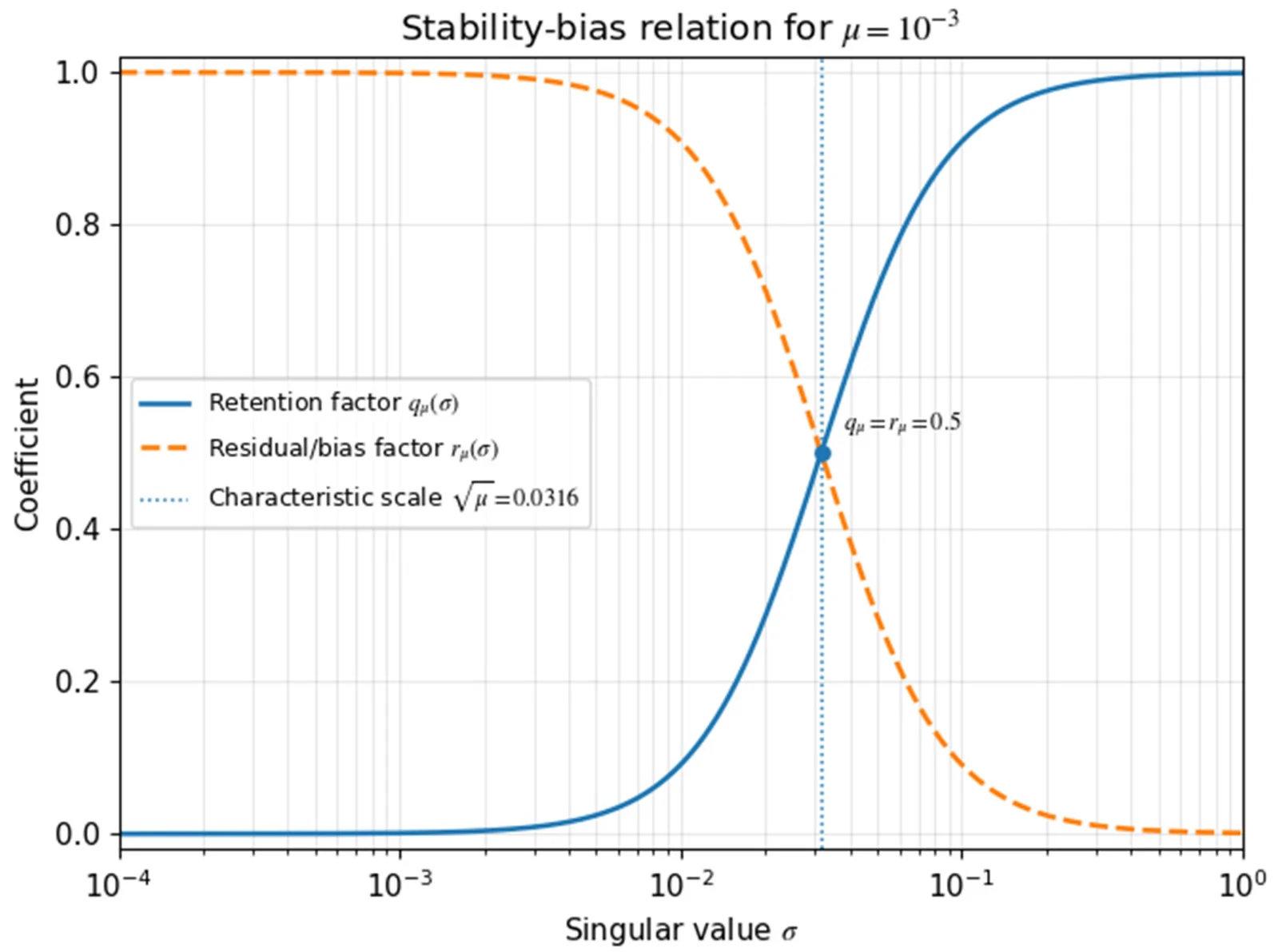
Stability–bias relationship of Tikhonov regularization for $\mu =10^{-3}$. The retention factor $q_{u}\left(\sigma \right)$ and the residual/bias factor $r_{\mu }\left(\sigma \right)$ are both equal to 0.5 at the characteristic scale $\sigma =\sqrt{\mu }$.

As shown in Figure 1, the standard inverse mapping 1/σ increases rapidly as the singular value decreases and therefore tends to amplify residuals and numerical errors along ill-conditioned directions. The Tikhonov filter function remains bounded near the origin and attains its maximum value of $1/(2\sqrt{\mu })$ at $\sigma =\sqrt{\mu }$. When the singular value is below the characteristic scale, the filter gain decreases with the singular value, significantly suppressing small-singular-value directions. When the singular value exceeds the characteristic scale, the filter function gradually approaches the standard inverse mapping. Figure 2 shows that the sum of the correction-retention coefficient and the residual-retention coefficient is always equal to 1. In the small-singular-value region, regularization effectively limits correction amplification, but the residual and solution biases along the corresponding directions are relatively large. In the large-singular-value region, the correction remains nearly unchanged, and the regularization bias is small. Therefore, selecting the regularization parameter essentially involves a trade-off between suppressing errors associated with ill-conditioning and preserving the accuracy of the Newton correction.

### 3.3. Chebyshev Polynomial Approximation of the Regularization Filter Function

QSVT requires constructing a polynomial P(z) over the interval $\left[-1,1\right]$ to approximate h(z). Because h(z) is an odd function, the approximating polynomial must also be chosen as an odd polynomial, as expressed in Equation (24):

(24) $$P(z)=\sum_{j=0}^{(d_{k}-1)/2}c_{2j+1,k}T_{2j+1}(z),$$

Here, $d_{k}$ denotes the polynomial degree used at the kth iteration and is chosen to be odd; $T_{2j+1}(z)$ is the Chebyshev polynomial of the first kind of degree 2j+1, and $c_{2j+1,k}$ denotes the corresponding expansion coefficient. Using an odd polynomial not only conforms to the symmetry of the target function but also satisfies the polynomial-parity requirement of QSVT-based singular-value transformation.

Define the maximum approximation error at the kth iteration as

(25) $$\varepsilon_{p,k}=\underset{z\in \left[-1,1\right]}{\max}\left|P(z)-h(z)\right|,$$

In addition to the approximation error, QSVT phase synthesis requires the polynomial to remain bounded over the entire interval, namely

(26) $$\left|P(z)\right|\leq 1,z\in \left[-1,1\right],$$

For the unregularized reciprocal function 1/z, the function diverges at the origin and can generally be approximated only over the interval $\left[-1,-\delta \right]\cup \left[\delta ,1\right]$, which excludes zero, where δ is related to the effective condition number of the matrix. In contrast, h(z) is continuous and bounded at the origin and can therefore be uniformly approximated over the full interval $\left[-1,1\right]$ without introducing an artificial singular-value cutoff interval. Consequently, the QSVT circuit no longer depends on an accurate prior estimate of the smallest nonzero singular value.

On the other hand, the regularization parameter still affects the polynomial degree. As μ decreases, $\gamma_{k}$ also decreases, causing the filter function to vary more sharply near the origin and requiring a higher-degree polynomial to achieve the same approximation error. As μ increases, the function becomes smoother, thereby reducing the difficulty of polynomial approximation. For sufficiently small $\gamma_{k}$, under the standard uniform-approximation criterion, the polynomial degree generally exhibits the following asymptotic scaling:

(27) $$d_{k}=O(\frac{1}{\gamma_{k}}\log\frac{1}{\varepsilon_{p,k}}),$$

The actual polynomial degree also depends on the coefficient-determination method, the stability of phase synthesis, and the error criterion employed.

**Figure 3:**
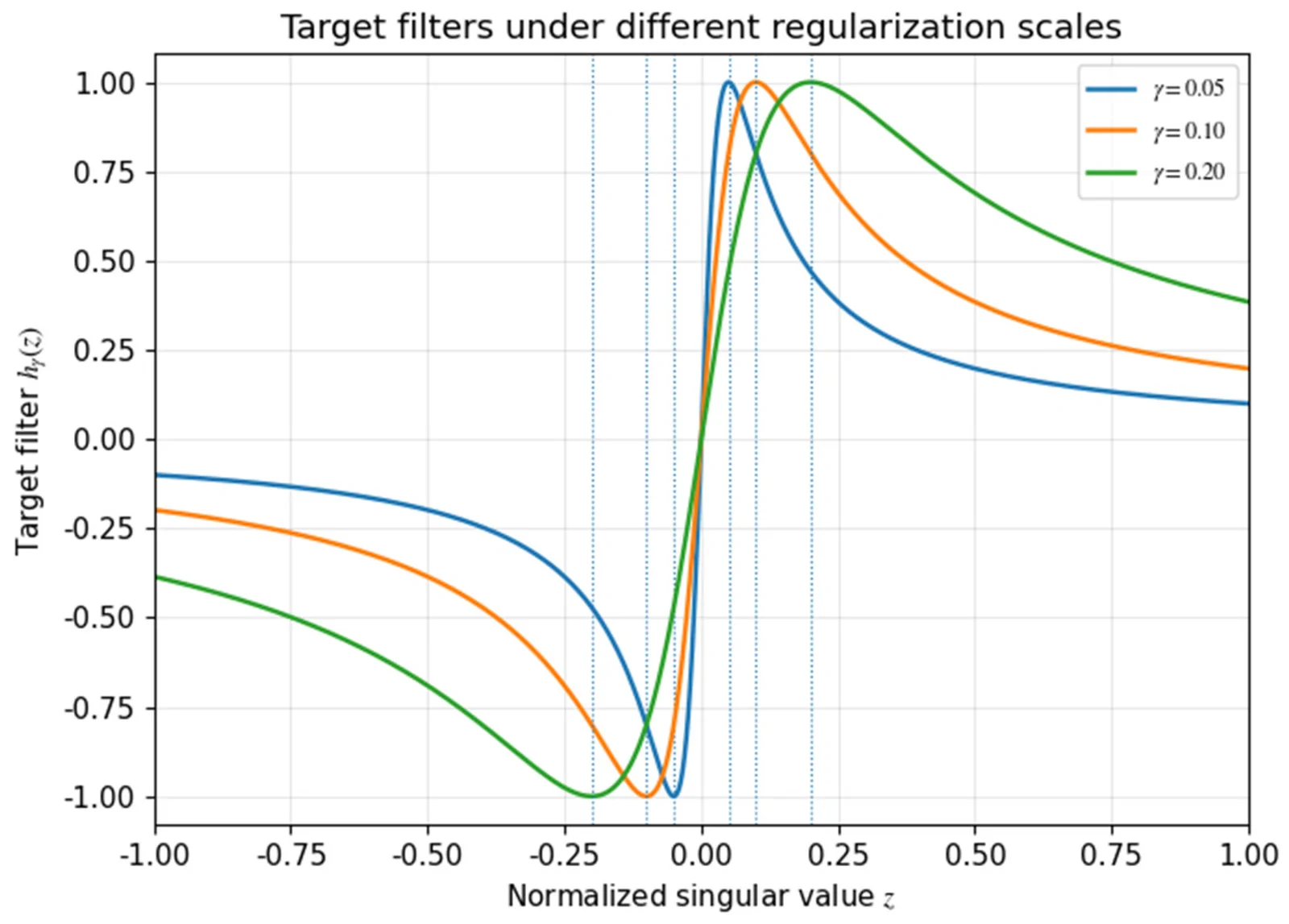
QSVT target filter functions for different regularization scales. As the regularization scale increases, the filter peak shifts toward larger normalized singular values, and the transition region broadens accordingly.

**Figure 4:**
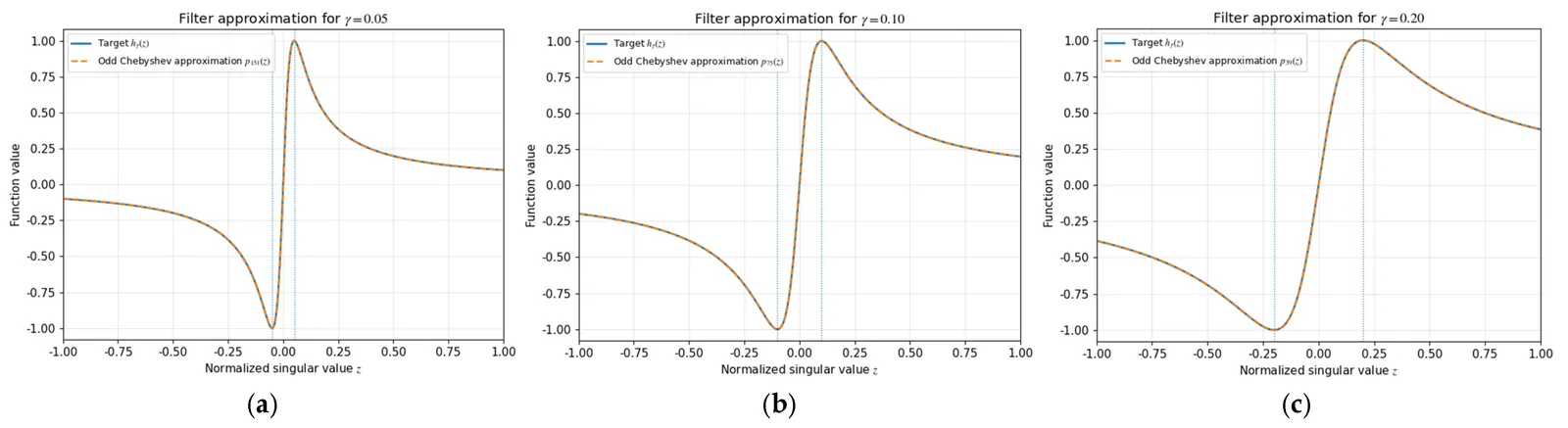
QSVT target filter functions and their odd Chebyshev polynomial approximations under different regularization scales: (**a**) γ=0.05; (**b**) γ=0.10; (**c**) γ=0.20.

**Figure 5:**
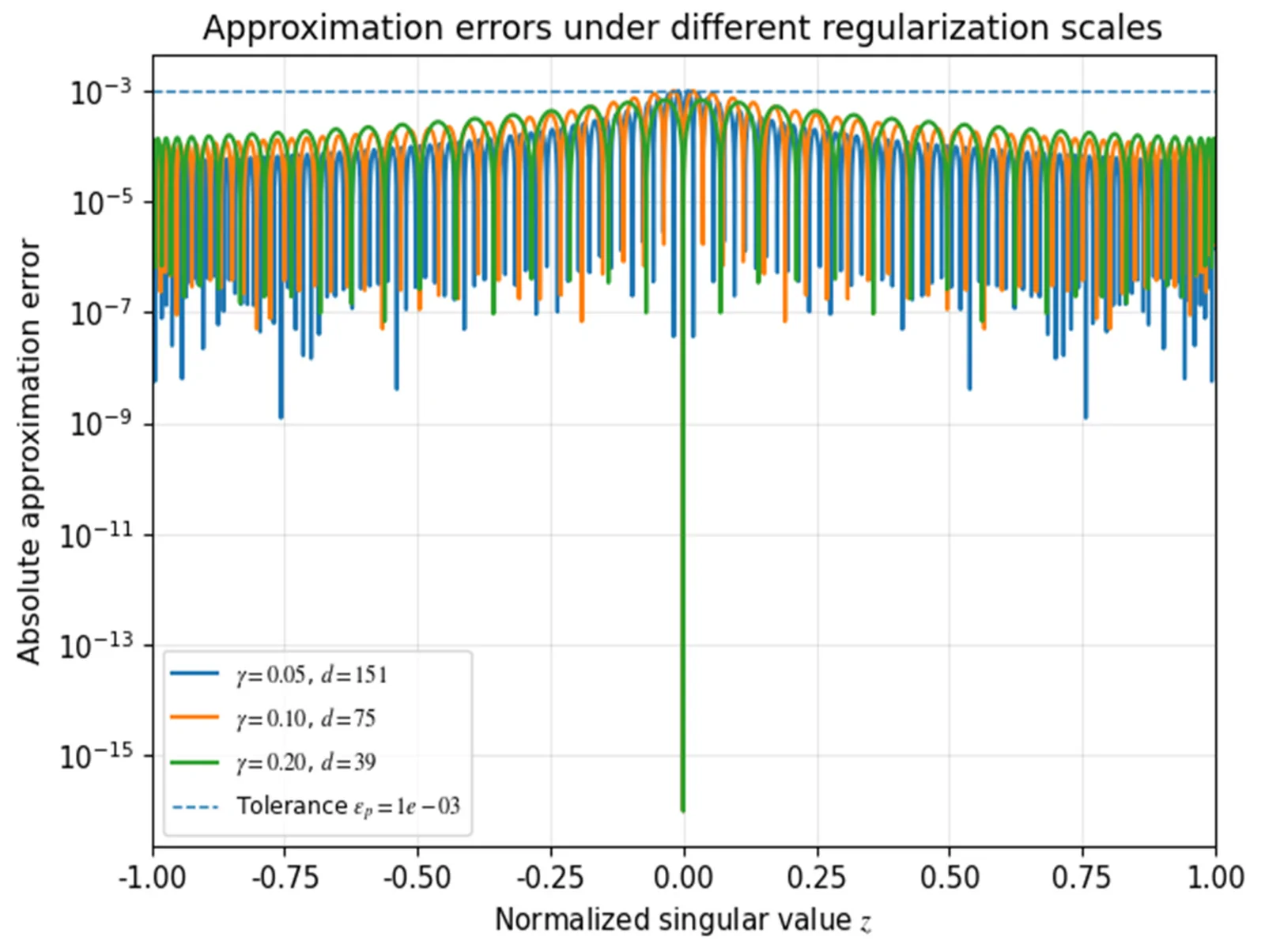
Absolute approximation errors of the QSVT filter functions under different regularization scales.

As shown in Figure 3, Figure 4 and Figure 5, the regularization scale $\gamma_{k}$ directly affects the local variation characteristics of the target filter function. When $\gamma_{k}$ is small, the extremum moves closer to the origin, the slope near the origin increases, and the filter curve exhibits a narrower transition region. To maintain a prescribed maximum uniform-approximation error, the degree of the odd Chebyshev polynomial must therefore be increased accordingly. As $\gamma_{k}$ decreases from 0.20 to 0.05, the minimum required odd degree increases from 39 to 151 under an error threshold of 10^−3^. This result indicates that although reducing the regularization scale decreases the regularization bias, it also increases the difficulty of QSVT polynomial approximation and the number of block-encoding calls. Therefore, the choice of the regularization parameter affects not only the numerical properties of the power-flow correction but also the quantum-circuit resource requirements. 

### 3.4. Regularized QSVT Correction-State Preparation and Power-Flow Iteration Procedure

Based on the polynomial coefficients obtained in Section 3.3, the QSVT phase sequence $\Phi_{k}=\left(\phi_{0,k},\phi_{1,k},\ldots ,\phi_{d_{k},k}\right)$ can be further generated, where $\phi_{j,k}$ denotes the jth phase-rotation parameter. The QSVT circuit implements the polynomial singular-value transformation by alternately invoking the block encoding of the Jacobian matrix, its conjugate transpose, and single-qubit phase rotations. Let the normalized Jacobian matrix $A_{k}$ be decomposed as $U_{k}\sum_{k}V_{k}^{T}$ according to Equation (4), after which the singular-value transformation is applied to $A_{k}^{T}$ The scaled power-mismatch vector is then normalized as $b_{k}=\frac{{\tilde{F}}_{k}}{{\left\|{\tilde{F}}_{k}\right\|}_{2}}$, and its corresponding quantum state is written as

(28) $$|b_{k}\rangle =\frac{1}{{\left\|{\tilde{F}}_{k}\right\|}_{2}}\sum_{i=1}^{n}{\tilde{F}}_{i,k}|i\rangle ,$$

Here, ${\tilde{F}}_{i,k}$ is the ith component of ${\tilde{F}}_{k}$, and $|i\rangle$ denotes the computational basis state. After preparing the residual state, the QSVT circuit constructed from the phase sequence $\Phi_{k}$ is applied. Under ideal block encoding and exact phase implementation, the branch corresponding to a successful ancilla qubit measurement represents the following vector:

(29) $$y_{p,k}=P^{\left(\sigma \right)}(A_{k}^{T})b_{k},$$

According to the scaling relationship between the bounded filter function and the original Tikhonov filter function, the regularized correction corresponding to the polynomial approximation is given by

(30) $$s_{p,k}=-\frac{{\left\|{\tilde{F}}_{k}\right\|}_{2}}{2\sqrt{\mu }}y_{p,k},$$

When the polynomial approximation and block encoding are error-free, $s_{p,k}$ coincides with the exact regularized correction $s_{k}^{*}$ defined in Section 2. Otherwise, the resulting correction is approximate and is denoted by ${\hat{s}}_{k}$. The corresponding physical-state correction is recovered using the state-scaling matrix $S_{x}$ according to Equation (31):

(31) $$\Delta {\hat{x}}_{k}=S_{x}{\hat{s}}_{k},$$

The bus-voltage phase angles and magnitudes are then updated according to Equation (3). After the update, the power-mismatch vector $F(x_{k+1})$ is recalculated. If condition ${\left\|F(x_{k+1})\right\|}_{\infty }\leq \varepsilon_{PF}$ is satisfied, the power-flow iteration terminates; otherwise, the next iteration is performed. Here, $\varepsilon_{PF}$ is the power-flow convergence tolerance, and ${\left\|\cdot \right\|}_{\infty }$ denotes the vector infinity norm.

### 3.5. Dynamic Quantum-Precision Configuration Under Regularization

The regularization parameter μ is held fixed, whereas the QSVT solution accuracy is dynamically adjusted throughout the power-flow iteration process. During the early iterations, the power-flow residual is relatively large, and a substantial discrepancy remains between the local linear model and the original nonlinear equations. Solving the quantum correction to very high accuracy at this stage therefore does not yield a proportional improvement in the outer iteration. As the iteration approaches the power-flow solution, however, the correction accuracy must be increased to prevent quantum errors from limiting the final convergence accuracy.

Let the actual approximate correction obtained by QSVT be ${\hat{s}}_{k}$. The error between ${\hat{s}}_{k}$ and the exact regularized correction $s_{k}^{*}$ can then be summarized as

(32) $${\left\|{\hat{s}}_{k}-s_{k}^{*}\right\|}_{2}\leq \frac{{\left\|{\tilde{F}}_{k}\right\|}_{2}}{2\sqrt{\mu }}\varepsilon_{Q,k},$$

Here, $\varepsilon_{Q,k}$ denotes the aggregate quantum implementation error at the kth iteration. This error comprises the polynomial-approximation error $\varepsilon_{p,k}$, the block-encoding error $\varepsilon_{BE,k}$, the residual-state preparation error, and the strategy-related and norm-recovery errors.

The residual of the regularized linear subproblem in Equation (14) of Section 2.4 can be rewritten as

(33) $$r_{k}^{q}=\left({\tilde{J_{k}}}^{T}\tilde{J_{k}}+\mu I_{n}\right)\left({\hat{s}}_{k}-s_{k}^{*}\right),$$

It then follows that

(34) $${\left\|r_{k}^{q}\right\|}_{2}\leq {\left\|{\tilde{J_{k}}}^{T}\tilde{J_{k}}+\mu I_{n}\right\|}_{2}\frac{{\left\|{\tilde{F}}_{k}\right\|}_{2}}{2\sqrt{\mu }}\varepsilon_{Q,k},$$

If the quantum-approximate correction is required to satisfy the inexact Newton condition ${\left\|r_{k}^{q}\right\|}_{2}\leq \eta_{k}{\left\|{\tilde{J_{k}}}^{T}\tilde{F_{k}}\right\|}_{2}$, the aggregate quantum error can be constrained according to Equation (35):

(35) $$\varepsilon_{Q,k}\leq \frac{2\sqrt{\mu }\eta_{k}{\left\|{\tilde{J_{k}}}^{T}\tilde{F_{k}}\right\|}_{2}}{{\left\|{\tilde{J_{k}}}^{T}\tilde{J_{k}}+\mu I_{n}\right\|}_{2}{\left\|\tilde{F_{k}}\right\|}_{2}},$$

Here, $\eta_{k}\in \left[0,1\right)$ is the inexact Newton forcing term at the kth iteration. Since ${\left\|{\tilde{J_{k}}}^{T}\tilde{J_{k}}+\mu I_{n}\right\|}_{2}\leq a_{k}^{2}+\mu$, a more readily computable sufficient condition can be adopted without explicitly constructing ${\tilde{J_{k}}}^{T}\tilde{J_{k}}+\mu I_{n}$:

(36) $$\varepsilon_{Q,k}\leq \frac{2\sqrt{\mu }\eta_{k}{\left\|{\tilde{J_{k}}}^{T}\tilde{F_{k}}\right\|}_{2}}{{\left\|a_{k}^{2}+\mu \right\|}_{2}{\left\|\tilde{F_{k}}\right\|}_{2}},$$

The forcing term can be determined from the outer power-flow residual as follows:

(37) $$\eta_{k}=\min\left\{\eta_{\max},c_{\eta }{\left(\frac{{\left\|{\tilde{F}}_{k}\right\|}_{2}}{{\left\|{\tilde{F}}_{0}\right\|}_{2}}\right)}^{\beta }\right\},$$

Here, $\eta_{\max}<1$ is the maximum allowable forcing term; $c_{\eta }>0$ is a proportionality coefficient; $\beta \in \left(0,1\right]$ controls the rate at which the accuracy requirement is tightened; and ${\tilde{F}}_{0}$ is the initial power residual. As the power-flow residual decreases, $\eta_{k}$ and the allowable aggregate quantum error decrease synchronously, thereby increasing the QSVT approximation degree and measurement precision.

## 4. Theoretical Properties and Quantum-Resource Analysis

This section further analyzes the descent condition of quantum-approximate correction, the composition of the total correction error, local convergence properties, and cumulative quantum-resource consumption based on the algorithmic framework established previously.

### 4.1. Descent Condition for the Quantum-Approximate Correction

For convenience in the subsequent analysis, we define

(38) $$H_{k}={\tilde{J_{k}}}^{T}\tilde{J_{k}}+\mu I_{n},g_{k}={\tilde{J_{k}}}^{T}\tilde{F_{k}},$$

Because μ>0, the matrix $H_{k}$ is symmetric positive definite. To determine whether the correction direction reduces the power mismatch, define the scaled power-mismatch objective function as

(39) $$\varphi (x)=\frac{1}{2}{\left\|\tilde{F}(x)\right\|}_{2}^{2},$$

At the current state $x_{k}$, the first-order directional derivative along the dimensionless correction direction s is given by

(40) $$\varphi^{\prime }(x_{k};s)=g_{k}^{T}s,$$

For the exact regularized correction, it follows that

(41) $$g_{k}^{T}s_{k}^{*}=-s_{k}^{*T}H_{k}s_{k}^{*}<0,$$

When $g_{k}\neq 0$ holds, $s_{k}^{*}$ is a strict descent direction for the current residual objective function. That is, although regularization modifies the spectral components of the standard Newton correction, it does not compromise the fundamental property that the correction direction reduces the power mismatch.

For the approximate correction ${\hat{s}}_{k}$ obtained from the actual QSVT computation, combining Equations (14) and (38) yields

(42) $${\hat{s}}_{k}=-H_{k}^{-1}g_{k}+H_{k}^{-1}r_{k}^{q},$$

Combining this result with Equation (41) yields

(43) $$g_{k}^{T}{\hat{s}}_{k}=-g_{k}^{T}H_{k}^{-1}g_{k}+g_{k}^{T}H_{k}^{-1}r_{k}^{q},$$

Using the matrix eigenvalue bounds, it follows that

(44) $$g_{k}^{T}{\hat{s}}_{k}\leq -\frac{{\left\|g_{k}\right\|}_{2}^{2}}{\lambda_{\max}(H_{k})}+\frac{{\left\|g_{k}\right\|}_{2}{\left\|r_{k}^{q}\right\|}_{2}}{\lambda_{\min}(H_{k})},$$

If the quantum-approximate correction satisfies the inexact Newton condition ${\left\|r_{k}^{q}\right\|}_{2}\leq \eta_{k}{\left\|g_{k}\right\|}_{2}$ given in Section 3, a sufficient condition ensuring that ${\hat{s}}_{k}$ remains a descent direction is

(45) $$\eta_{k}<\frac{\lambda_{\min}(H_{k})}{\lambda_{\max}(H_{k})}=\frac{1}{k_{2}(H_{k})},$$

Here, $k_{2}(H_{k})$ denotes the two-norm condition number of $H_{k}$. Taking $\lambda_{\min}(H_{k})\geq \mu ,\lambda_{\max}(H_{k})\leq a_{k}^{2}+\mu$ into account, the following more readily computable condition may also be adopted:

(46) $$\eta_{k}<\frac{\mu }{a_{k}^{2}+\mu },$$

This condition indicates that the quantum-solve accuracy cannot be specified independently of the regularized model. When the regularization parameter is small or the Jacobian matrix has a broad spectral range, the residual of the linear subproblem must be constrained more stringently to ensure that the quantum correction remains a well-defined descent direction.

### 4.2. Regularization Bias, Quantum Error, and Local Convergence

The discrepancy between the regularized QSVT correction and the standard Newton correction consists primarily of the regularization bias and the quantum implementation error, as expressed in Equation (47):

(47) $${\hat{s}}_{k}-s_{k}^{N}=(s_{k}^{*}-s_{k}^{N})+({\hat{s}}_{k}-s_{k}^{*}),$$

Here, $s_{k}^{N}$ denotes the standard Newton correction, and $s_{k}^{*}$ denotes the exact regularized correction. When the scaled Jacobian matrix ${\tilde{J}}_{k}$ is nonsingular, the standard Newton correction is given by

(48) $$s_{k}^{N}=-{\tilde{J}}_{k}^{-1}{\tilde{F}}_{k},$$

From the singular-value representations of the two corrections, it follows that

(49) $${\left\|s_{k}^{*}-s_{k}^{N}\right\|}_{2}\leq \frac{\mu }{{\tilde{\sigma }}_{\min,k}({\tilde{\sigma }}_{\min,k}^{2}+\mu )}{\left\|{\tilde{F}}_{k}\right\|}_{2},$$

Here, ${\tilde{\sigma }}_{\min,k}$ is the smallest singular value of $\tilde{J_{k}}$. This expression shows that the regularization bias increases with μ and becomes more pronounced as the Jacobian matrix approaches singularity. Combining this result with Equation (32) yields the following upper bound on the total error of the quantum-approximate correction relative to the standard Newton correction:

(50) $${\left\|s_{k}^{*}-s_{k}^{N}\right\|}_{2}\leq \left[\frac{\mu }{{\tilde{\sigma }}_{\min,k}({\tilde{\sigma }}_{\min,k}^{2}+\mu )}+\frac{\varepsilon_{Q,k}}{2\sqrt{\mu }}\right]{\left\|{\tilde{F}}_{k}\right\|}_{2},$$

This expression clearly reveals the trade-off: increasing the regularization bias reduces the amplification of quantum errors, whereas decreasing the regularization bias increases sensitivity to quantum errors. Therefore, a smaller regularization parameter is not necessarily better, nor does a larger value necessarily provide greater stability. Its value should be selected by jointly considering the degree of ill-conditioning, the required accuracy of the power-flow correction, and the practically achievable error level of the quantum circuit.

To analyze local convergence, let $x^{*}$ denote a solution of the power-flow equations, and define the scaled state error as

(51) $$y_{k}=S_{x}^{-1}(x_{k}-x^{*}),$$

Assume that, in a neighborhood of $x^{*}$, the scaled power-flow function is continuously differentiable, the scaled Jacobian matrix is locally Lipschitz continuous, $\tilde{J}(x^{*})$ is nonsingular, and the regularization parameter μ>0 remains fixed. Neglecting higher-order terms, the local error-propagation matrix of the exact regularized iteration is given by

(52) $$M_{\mu ,*}=\mu {\left({\tilde{J_{*}}}^{T}\tilde{J_{*}}+\mu I_{n}\right)}^{-1},$$

Define the local regularized convergence factor as

(53) $$\rho_{\mu ,*}:={\left\|M_{\mu ,*}\right\|}_{2}=\frac{\mu }{{\tilde{\sigma }}_{\min,*}^{2}+\mu },$$

Because $0<\rho_{\mu ,*}<1$, the exact regularized iteration exhibits local linear convergence in a neighborhood of the solution. After accounting for the quantum-approximation error, the local state error satisfies

(54) $${\left\|y_{k+1}\right\|}_{2}\leq \left[\rho_{\mu ,*}+\frac{a_{*}}{2\sqrt{\mu }}\varepsilon_{Q,k}\right]{\left\|y_{k}\right\|}_{2}+C{\left\|y_{k}\right\|}_{2}^{2},$$

Here, $a_{*}$ is the normalization factor of the Jacobian matrix in a neighborhood of the solution, and C>0 is a constant determined by the power-flow nonlinearity and the local variation in the Jacobian matrix. Therefore, if condition $\rho_{\mu ,*}+\frac{a_{*}}{2\sqrt{\mu }}\varepsilon_{Q,k}<1$ is satisfied at the solution, the quantum-approximate iteration retains local convergence. If the dynamic-accuracy allocation drives the aggregate quantum error to zero, its asymptotic convergence factor approaches $\rho_{\mu ,*}=\frac{\mu }{{\tilde{\sigma }}_{\min,*}^{2}+\mu }$.

A fixed regularization parameter alters the linearized mapping of the Newton iteration at the solution point. Therefore, this study establishes only a local linear convergence result. When μ is sufficiently small relative to ${\tilde{\sigma }}_{\min,*}^{2}$, $\rho_{\mu ,*}$ approaches zero, and the convergence rate can approach that of the standard Newton method. However, the ability of regularization to suppress ill-conditioned directions and quantum errors is correspondingly weakened.

The local linear convergence established the above results from retaining a fixed positive regularization parameter. A faster asymptotic rate can be recovered if the regularization parameter is allowed to decrease as the nonlinear residual approaches zero. Let $\mu_{k}$ denote an iteration-dependent regularization parameter. In a neighborhood of a nonsingular solution $x^{*}$, the difference between the regularized correction and the exact Newton correction is of order $O\left(\mu_{k}\left\|x_{k}-x^{*}\right\|\right)$.

Consequently, the local error recursion can be written in the form $\left\|x_{k+1}-x^{*}\right\|\leq C_{1}{\left\|x_{k}-x^{*}\right\|}^{2}+C_{2}\mu_{k}\left\|x_{k}-x^{*}\right\|+C_{3}\left\|x_{k}-x^{*}\right\|$, where the last term represents the effect of the inexact quantum correction through the forcing term $\eta_{k}$. Therefore, $\mu_{k}\to 0$ and $\eta_{k}\to 0$ are sufficient for the regularization and inexact-solve perturbations to vanish asymptotically, allowing superlinear convergence. Moreover, because $\left\|F_{k}\right\|=O\left(\left\|x_{k}-x^{*}\right\|\right)$ near a nonsingular solution, choosing $\mu_{k}=O\left(\left\|F_{k}\right\|\right)$ and $\eta_{k}=O\left(\left\|F_{k}\right\|\right)$ makes both perturbation terms second order, so that the local quadratic convergence rate of the Newton method can in principle be recovered.

It should also be noted that decreasing $\mu_{k}$ is not computationally free in the QSVT implementation. According to $\gamma_{k}=\sqrt{\mu_{k}}/a_{k}$, a smaller $\mu_{k}$ produces a smaller normalized regularization scale and generally requires a higher polynomial degree to approximate the increasingly sharp filter function. Thus, an adaptive decay of $\mu_{k}$ introduces an additional trade-off between asymptotic Newton convergence and quantum-circuit depth. The present work therefore retains a fixed μ and leaves joint adaptation of $\mu_{k}$ and quantum precision for future investigation.

### 4.3. Cumulative Quantum Resources Under Dynamic-Accuracy Allocation

The procedure for determining the QSVT polynomial degree $d_{k}$ has been described in Section 3. This section primarily evaluates the effect of variations in this degree on the resources required for multi-iteration power-flow computation. At the kth iteration, the number of calls made by QSVT to the block-encoding unitary and its adjoint is linearly related to the polynomial degree and can be expressed as

(55) $$Q_{BE,k}=c_{q}d_{k}+O(1),$$

Here, $c_{q}$ is a constant determined by the specific QSVT circuit convention. If the circuit depth of a single block-encoding call is $D_{BE,k}$, the depth of the main QSVT circuit can be approximated as

(56) $$D_{QSVT,k}=O(d_{k}D_{BE,k}),$$

Assuming that the power-flow computation requires a total of K outer iterations, the cumulative polynomial degree under the dynamic-accuracy strategy is given by

(57) $$C_{d}=\sum_{k=0}^{K-1}d_{k},$$

The cumulative number of block-encoding calls is given by

(58) $$C_{BE}=\sum_{k=0}^{K-1}Q_{BE,k},$$

If the fixed-accuracy method employs the highest polynomial degree required to satisfy the final accuracy criterion, namely $d_{fit}=\underset{0\leq k<K}{\max}d_{k}$, throughout all iterations, the corresponding cumulative polynomial degree is given by

(59) $$C_{d}^{fix}=Kd_{fix},$$

Accordingly, the cumulative polynomial degree reduction ratio of the dynamic-accuracy strategy is defined as

(60) $$R_{d}=1-\frac{C_{d}}{C_{d}^{fix}}=1-\frac{\sum_{k=0}^{K-1}d_{k}}{Kd_{fix}},$$

When the block-encoding structure remains approximately identical across iterations, $R_{d}$ can also be used as an approximate indicator of the reduction ratio in block-encoding calls and the depth of the main QSVT circuit. If the postselection success probability of the ancillary qubits at each iteration is $p_{s,k}$, and amplitude amplification is applied, the effective cumulative query complexity can be further expressed as

(61) $$C_{eff}=O\left(\sum_{k=0}^{K-1}\frac{d_{k}}{\sqrt{p_{s,k}}}\right),$$

This metric reflects the effect of the filter-output amplitude on the actual number of circuit executions. The total qubit count is primarily determined by the system register, the ancillary registers required for block encoding, and the state preparation registers. For a power-flow correction problem of dimension n, the system register requires at least $\log_{2}n$ qubits. Because dynamic-accuracy allocation mainly changes the polynomial degree without altering the matrix dimension, its principal advantage lies in reducing the number of block-encoding calls and the circuit depth rather than the qubit count. The subsequent case studies evaluate the stability, solution accuracy, and quantum-resource requirements of the proposed method using the descent condition, total-error decomposition, and cumulative resource metrics defined in this section.

### 4.4. End-to-End Complexity Including Classical Readout

Equation (61) characterizes the effective query complexity required to prepare the QSVT correction state, including the effect of postselection and amplitude amplification. This quantity is useful for comparing different quantum-precision allocation strategies because the polynomial degree directly determines the number of block-encoding calls. However, it should not be interpreted as the end-to-end computational complexity of the complete hybrid power-flow algorithm. In the proposed framework, the nonlinear power-flow iteration and state update remain classical. Therefore, after the QSVT circuit prepares a quantum state proportional to the regularized correction, sufficient classical information must be extracted to recover the correction vector, update the voltage state, and evaluate the power mismatch and Jacobian matrix for the next iteration. The costs associated with state preparation, block encoding, quantum-state readout, and classical postprocessing must therefore be considered together when assessing a possible computational advantage over classical sparse power-flow solvers.

Let $C_{BE,k}$ denote the implementation cost of one call to the block-encoding unitary or its adjoint at the kth iteration, and let $C_{sp,k}$ denote the cost of preparing the normalized power-mismatch state $|b_{k}\rangle$. Since the QSVT circuit invokes the block encoding a number of times proportional to the polynomial degree $d_{k}$, the cost of preparing one successful copy of the correction state can be written, up to convention-dependent constant and logarithmic factors, as

(62) $$C_{state,k}=\tilde{O}\left[\frac{d_{k}C_{BE,k}+C_{sp,k}}{\sqrt{p_{s,k}}}\right],$$

where $p_{s,k}$ is the postselection success probability and the factor $1/\sqrt{p_{s,k}}$ corresponds to amplitude amplification. Equation (62) reduces to the query-count expression in Equation (61) when the costs of state preparation and individual block-encoding calls are treated as unit costs. In an actual implementation, however, $C_{BE,k}$ and $C_{sp,k}$ depend on the adopted matrix-access and data-loading models. They should therefore be retained explicitly in an end-to-end resource estimate.

The output requirement introduces an additional cost that is absent from Equation (61). The successful QSVT branch encodes the direction of the regularized correction in quantum amplitudes, whereas the classical outer iteration requires the components of $\Delta x_{k}=S_{x}s_{k}$ to update the voltage magnitudes and phase angles. Consequently, the complete correction vector must be reconstructed to a prescribed readout accuracy unless the outer iteration is redesigned to retain the state in quantum form. Let $\varepsilon_{ro,k}$ denote the allowable error associated with classical reconstruction of the normalized correction state, and let $N_{ro,k}$ denote the corresponding number of successfully prepared quantum states required by the readout procedure. For full reconstruction of an n-dimensional pure state, representative tomography-type scaling is

(63) $$N_{ro,k}=\tilde{O}\left(\frac{n}{\varepsilon_{ro,k}^{2}}\right),$$

where logarithmic factors and protocol-dependent constants are omitted. The exact scaling depends on the measurement strategy and the adopted error metric; Equation (63) is therefore used here as a representative full-vector readout model rather than as a universal implementation cost. Independently of the tomography protocol, explicitly producing an n-component classical correction vector requires at least $\Omega \left(n\right)$ classical output operations. Thus, the logarithmic number of qubits required to represent the correction state does not by itself imply a logarithmic end-to-end dependence on the power-flow dimension.

In addition to the normalized correction direction, its physical magnitude must be recovered before the state-variable update can be performed. This step may be implemented through success-probability estimation, amplitude estimation, repeated sampling, or another norm-recovery procedure. Let $C_{norm,k}$ denote the corresponding cost. The associated norm-recovery error is already included in the aggregate quantum implementation error introduced in Section 3.5. Accordingly, both $\varepsilon_{ro,k}$ and the norm-recovery accuracy should be selected consistently with the admissible aggregate error $\varepsilon_{Q,k}$ imposed by Equations (35) and (36). In particular, the readout accuracy need not remain uniformly high throughout all outer iterations. A relatively coarse reconstruction can be permitted when the power-flow residual and forcing term are large, whereas a more accurate correction must be recovered near the solution. Therefore, the residual-controlled accuracy principle used for QSVT polynomial approximation can also be applied to the readout stage.

Let $C_{asm,k}$ denote the classical cost of evaluating the nonlinear power mismatch and assembling the scaled Jacobian matrix, $C_{data,k}$ the cost associated with constructing or updating the data structures required for block encoding, and $C_{upd,k}$ the cost of reconstructing the physical correction and updating the classical power-flow state. Under a full-vector readout architecture, the end-to-end complexity of the proposed hybrid algorithm over K outer iterations can then be expressed as

(64) $$C_{E2E}^{Q}=\sum_{k=0}^{K-1}\left[C_{asm,k}+C_{data,k}+N_{ro,k}C_{state,k}+C_{norm,k}+C_{upd,k}\right].$$

Substituting Equations (62) and (63) into Equation (64) shows that the contribution associated with reconstructing the correction vector has the representative scaling

(65) $$C_{out,k}=\tilde{O}\left[\frac{n}{\varepsilon_{ro,k}^{2}}\frac{d_{k}C_{BE,k}+C_{sp,k}}{\sqrt{p_{s,k}}}\right]+C_{norm,k}.$$

Equation (65) makes it explicit that a reduction in the QSVT polynomial degree does not translate directly into the same reduction in end-to-end runtime. In particular, if a newly prepared QSVT output state is required for each measurement sample, the cost of correction-state preparation is repeated throughout the readout process. Hence, full-vector extraction can become comparable to or larger than the QSVT itself, especially when n is large or when a small $\varepsilon_{ro,k}$ is required near convergence.

For comparison, consider a classical Newton-type power-flow implementation in which the same mismatch and Jacobian are assembled, and the linearized correction equation is solved using a sparse direct or iterative method. Its end-to-end complexity may be written generically as

(66) $$C_{E2E}^{C}=\sum_{k=0}^{K_{C}-1}\left[C_{asm,k}^{C}+C_{solve,k}^{C}+C_{upd,k}^{C}\right],$$

where $C_{solve,k}^{C}$ represents the sparse linear-solver cost. No single asymptotic expression applies to all power networks because this cost depends on the sparsity pattern, fill-in, ordering strategy, conditioning, preconditioning, and solver type. For a Krylov-type method, for example, the dominant matrix-vector operations scale with the number of nonzero Jacobian entries and the required number of inner iterations, whereas sparse factorization costs depend strongly on fill-in. A fair comparison should therefore use the actual classical sparse solver appropriate to the network under study rather than a dense-matrix complexity bound.

If the classical and quantum formulations require approximately the same number of outer iterations and share comparable Jacobian assembly and state-update costs, a necessary condition for an end-to-end quantum advantage can be stated as

(67) $$\sum_{k=0}^{K-1}\left[C_{data,k}+N_{ro,k}C_{state,k}+C_{norm,k}\right]<\sum_{k=0}^{K-1}C_{solve,k}^{C}.$$

When the numbers of outer iterations differ, the complete expressions in Equations (64) and (66) must instead be compared directly. Equation (67) provides an advantage criterion rather than a universal crossover system size. A numerical crossover point cannot be determined from the system dimension alone because it also depends on the matrix-access model, block-encoding implementation, postselection probability, requested output accuracy, classical sparsity structure, and hardware-level gate costs. This observation is consistent with recent end-to-end analyses of quantum power-flow algorithms, which show that the input-access model, conditioning, and classical readout requirement can fundamentally alter the practical advantage predicted from the quantum linear-solver complexity alone.

This distinction is particularly relevant to the present method. Residual-controlled dynamic precision reduces $d_{k}$ during the early outer iterations and therefore directly reduces the number of block-encoding calls required by the QSVT kernel. It can also reduce readout cost if $\varepsilon_{ro,k}$ is relaxed consistently with the inexact Newton forcing term during these iterations. Nevertheless, because the present hybrid architecture explicitly updates all voltage magnitudes and phase angles in classical form, full-vector readout cannot be omitted from an end-to-end implementation. Consequently, the principal result of the present resource analysis is a reduction in the quantum computational kernel relative to a fixed-accuracy QSVT implementation, rather than a demonstrated end-to-end speedup over a state-of-the-art classical sparse power-flow solver.

For the 69-bus system considered in Section 5, the state dimension is n=163. The reported reduction in the cumulative polynomial degree therefore quantifies the reduction in QSVT polynomial and block-encoding-query requirements under the same quantum power-flow architecture. The result should not be interpreted as an equal percentage reduction in total execution time or as a corresponding speedup relative to the classical solution. Establishing such a practical crossover would additionally require a hardware-specific block-encoding construction, state preparation implementation, a fault-tolerant gate model, readout protocol, and benchmark against an optimized classical sparse solver. These aspects are beyond the scope of the present work and constitute important directions for subsequent implementation-oriented studies.

## 5. Case Study

To evaluate the solution accuracy and quantum-resource allocation of the proposed residual-controlled, regularized quantum singular-value transformation-based inexact Newton power-flow method under ill-conditioned conditions, this section conducts case studies on a 69-bus radial distribution system [34]. The comparison methods include the classical solution, the regularized Newton–Raphson method (RNR), the fixed-accuracy regularized QSVT power-flow method (FP-RQSVT), and the proposed residual-controlled, regularized QSVT-based inexact Newton power-flow method (RC-RQSVT-INPF).

### 5.1. Test System and Parameter Settings

The test case employs a 69-bus radial distribution system with a system base power of 10 MVA and a base voltage of 12.7 kV. Bus 1 is designated as the slack bus, while the remaining 68 buses are PQ buses. Accordingly, the polar-coordinate power-flow model contains 68 bus-voltage phase-angle variables and 68 bus-voltage-magnitude variables, yielding a state-vector dimension of 136. The system consists of 68 in-service radial branches, with the slack source connected only at Bus 1. Because the branch impedances span a wide range and the loads are distributed along a long feeder, the power-flow Jacobian matrix exhibits a broad singular-value spectrum. The system is therefore suitable for evaluating correction-error amplification under ill-conditioned conditions and the effectiveness of regularized filtering. The unified parameter settings are listed in Table 1.

In the case study, the block-encoding normalization factor is fixed at $a_{k}=1.7$, which upper bounds the spectral norm of the scaled Jacobian over all iterations. With $\mu =3\times 10^{-9}$, Equation (20) therefore gives $\gamma_{k}=3.2219\times 10^{-5}$.

### 5.2. Power-Flow Solution Results

**Figure 6:**
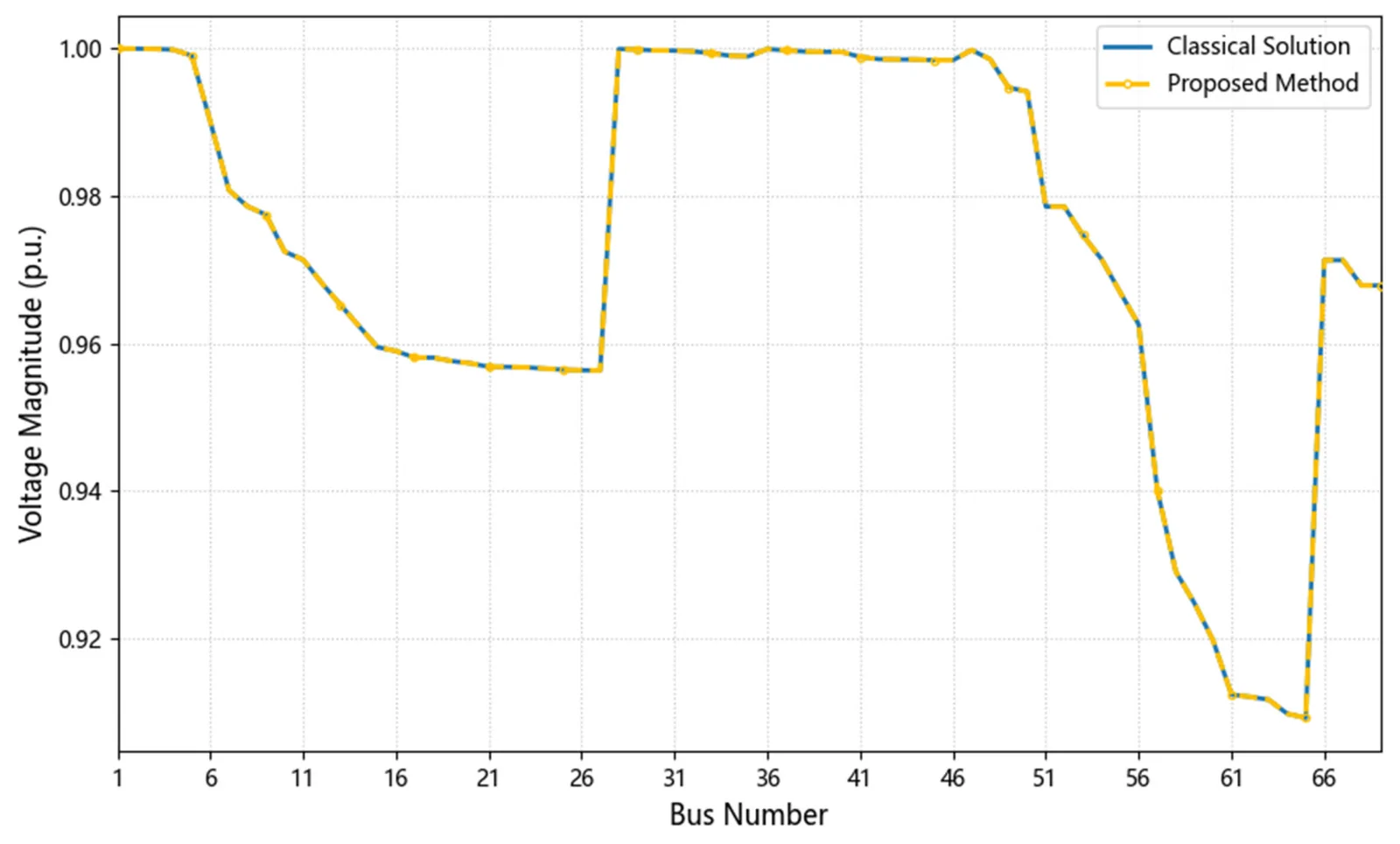
Comparison of voltage magnitudes between the classical solution and the proposed method.

**Figure 7:**
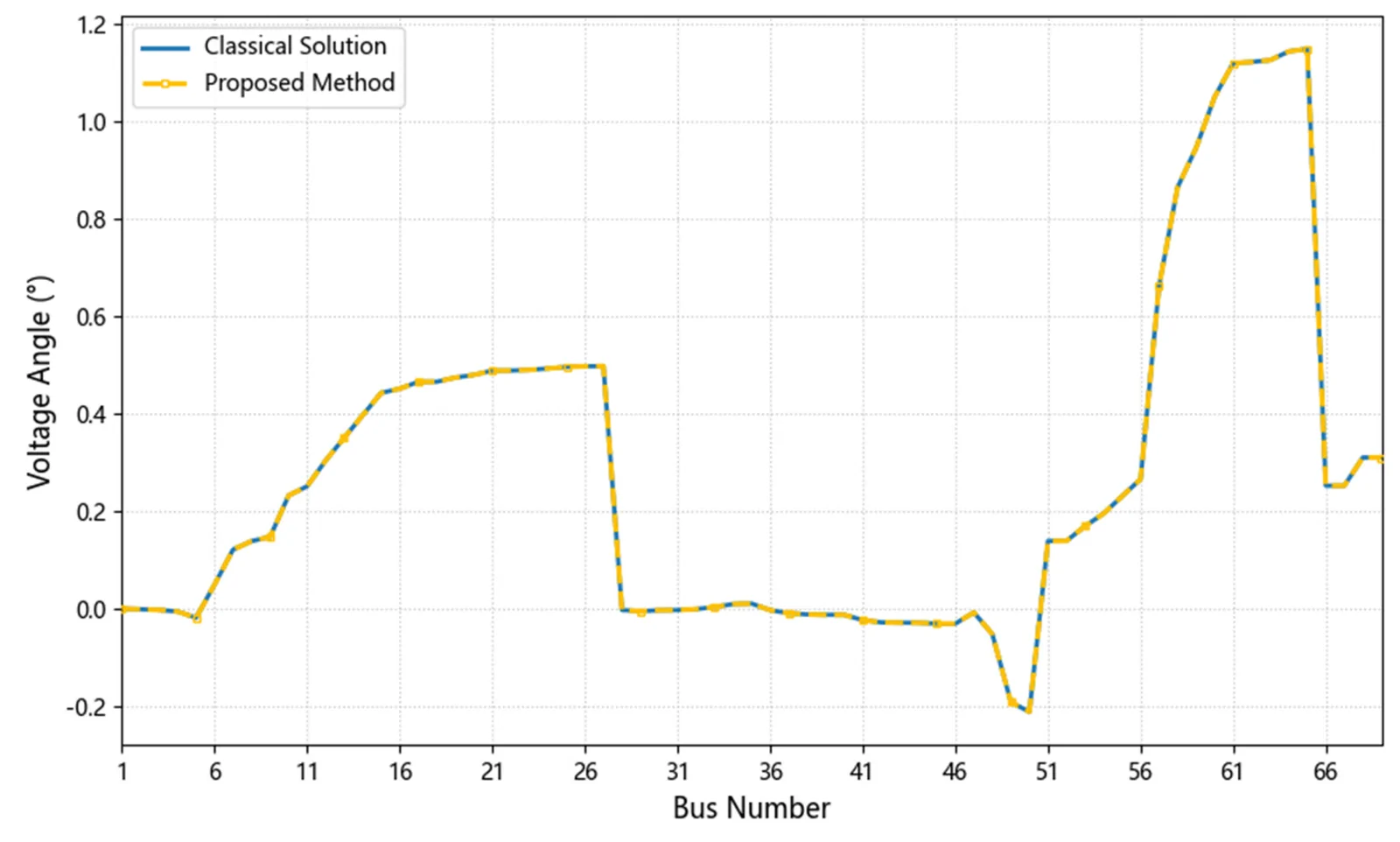
Comparison of voltage phase angles between the classical solution and the proposed method.

The bus-voltage magnitudes and phase angles relative to the classical solution are shown in Figure 6 and Figure 7, respectively.

The bus-voltage-magnitude and phase-angle profiles obtained using the proposed method almost completely overlap those of the classical solution, thereby verifying the correctness of the proposed method. The maximum bus errors of the three regularized methods relative to the classical solution are presented in Table 2.

The voltage-magnitude errors of all three methods are below $6\;\;\times \;\;10^{-8}\;p.u.$, and their voltage-angle errors are below ${8\;\;\times \;\;10^{-7}}^{\circ }$. Although the proposed method adopts dynamically adjusted QSVT accuracy, its final error does not exceed that of the fixed-accuracy method. This indicates that reducing the accuracy of the quantum linear solve during the early iterations does not necessarily degrade the accuracy of the final power-flow solution.

### 5.3. Residual-Controlled QSVT Accuracy and Resource Analysis

The proposed method selects the QSVT accuracy according to the forcing term $\eta_{k}$ and the actual residual ratio of the regularized linear subproblem. The QSVT approximation accuracy and corresponding polynomial degree at each stage are presented in Table 3.

During the first three iterations, the outer residual remains relatively large, and the forcing terms are 0.900, 0.415, and 0.191, respectively. Therefore, a QSVT polynomial with an approximation error of 0.3 is sufficient to satisfy the inexact Newton condition. As the power-flow residual decreases, the forcing term is progressively reduced, and the required QSVT approximation error is successively tightened to 0.1, 0.03, 0.01, 0.003, 0.001, and 0.0003.

Based on the iteration-dependent polynomial degrees reported in Table 3, the cumulative polynomial degree of the fixed-accuracy QSVT method is 5,605,054, whereas that of the proposed method is 2,571,585. Therefore, the reduction in the cumulative polynomial degree achieved by the proposed method relative to fixed-accuracy QSVT is given by

(68) $$R_{d}=1-\frac{2,571,585}{5,605,054}\times 100\%=54.12\%,$$

Because the QSVT polynomial degree is approximately linear in the number of block-encoding calls, the cumulative block-encoding-call proxy is likewise reduced by 54.12% when convention-dependent constant factors are neglected. At the level of the QSVT polynomial degree and block-encoding-call proxy, this result demonstrates that the residual-control strategy effectively avoids repeated calls to fixed high-accuracy QSVT during the early iterations, thereby providing a more rational resource-allocation scheme for the quantum solution of ill-conditioned AC power-flow problems.

## 6. Conclusions

To address the amplification of correction errors caused by small singular values of the Jacobian matrix in ill-conditioned AC power-flow problems, as well as the redundant quantum-circuit resources incurred by fixed high-accuracy quantum solves, this study proposes a residual-controlled, regularized QSVT-based inexact Newton power-flow method. The method first applies dimensionless scaling to the state variables and power mismatches and formulates the Newton correction as a Tikhonov-regularized least-squares problem to suppress anomalous corrections along ill-conditioned directions. It then approximates the regularized singular-value filter function using a bounded odd Chebyshev polynomial and applies the resulting transformation directly to the scaled Jacobian matrix through QSVT, thereby avoiding the explicit construction of the normal equations and the associated squared-condition-number effect.

For the allocation of quantum-solve accuracy, the residual of the regularized linear subproblem is adopted as the accuracy criterion. The inexact Newton forcing term, the QSVT approximation error, and the polynomial degree are dynamically adjusted according to the residual of the outer power-flow iteration. Lower accuracy is used during the early iterations to reduce unnecessary quantum-circuit overhead, whereas the accuracy is progressively increased as the iteration approaches the power-flow solution, thereby balancing outer iteration convergence against inner-solve resource consumption. Theoretical analysis shows that the regularized correction remains bounded, that a quantum-approximate correction satisfying the prescribed residual condition retains a descent direction, and that the dynamic-accuracy strategy primarily reduces the cumulative number of block-encoding calls and the circuit depth without changing the required number of qubits.

The reported 54.12% reduction therefore refers specifically to the cumulative QSVT polynomial degree and block-encoding-call proxy relative to the fixed-accuracy QSVT implementation. It should not be interpreted as a 54.12% reduction in end-to-end execution time relative to a classical power-flow solver. Full computational advantage additionally depends on block-encoding construction, quantum-state preparation, postselection, fault-tolerant circuit cost, and classical readout of the correction vector.

## Figures and Tables

**Table 1:** Unified parameters for the power-flow test cases.

Parameter	Value
Power-Flow Convergence Tolerance	10^−8^
Maximum Number of Iterations	100
Regularization Parameter	3 × 10^−9^
Block-Encoding Normalization Factor	1.7
Regularization Scale	3.2219 × 10^−5^
Fixed-Accuracy QSVT Approximation Error	10^−5^
Upper Bound of the Forcing Term	0.9
Lower Bound of the Forcing Term	10^−4^
Forcing-Term Exponent	0.5

**Table 2:** Maximum bus errors of the regularized methods relative to the classical solution.

Method	Maximum Voltage-Magnitude Error (p.u.)	Maximum Voltage-Angle Error (°)
RNR	5.9247 × 10^−8^	7.8657 × 10^−7^
FP-RQSVT	5.9250 × 10^−8^	7.8661 × 10^−7^
RC-RQSVT-INPF	3.8002 × 10^−8^	5.0526 × 10^−7^

**Table 3:** Dynamic accuracy configuration for RC-RQSVT-INPF.

Outer Iteration Number	QSVT Approximation Error	Polynomial Degree
0——2	0.3	75,351
3——4	0.1	112,901
5——6	0.03	151,393
7——8	0.01	185,805
9——12	0.003	223,283
13	0.001	257,411
14	0.0003	294,791

## Data Availability

The original contributions presented in this study are included in the article. Further inquiries can be directed to the corresponding author.
